# An analysis of core EPAs reveals a gap between curricular expectations and medical school graduates’ self-perceived level of competence

**DOI:** 10.1186/s12909-021-02534-w

**Published:** 2021-02-16

**Authors:** Adrian Marty, Sonia Frick, Heidi Bruderer Enzler, Sabine Zundel

**Affiliations:** 1 Institute of Anaesthesiology, University Hospital, Zurich, Switzerland; 2 Internal Medicine, Spital Limmattal, Schlieren, Switzerland; 3 Dean’s Office, Faculty of Medicine, University of Zurich, Zurich, Switzerland; 4 Department of Paediatric Surgery, Children’s Hospital, Lucerne, Switzerland

**Keywords:** Competency-based medical education, Entrustable professional activities, Undergraduate medical education, Self-assessment

## Abstract

**Background:**

Entrustable Professional Activities (EPAs) are being implemented worldwide as a means to promote competency-based medical education. In Switzerland, the new EPA-based curriculum for undergraduate medical education will be implemented in 2021. The aim of our study was to analyze the perceived, self-reported competence of graduates in 2019. The data represent a pre-implementation baseline and will provide guidance for curriculum developers.

**Methods:**

Two hundred eighty-one graduates of the Master of Human Medicine program of the University of Zurich who had passed the Federal Licensing Exam in September 2019 were invited to complete an online survey. They were asked to rate their needed level of supervision (“observe only”, “direct, proactive supervision”, “indirect, reactive supervision”) for 46 selected EPAs. We compared the perceived competence with the expected competence of the new curriculum.

**Results:**

The response rate was 54%. The need for supervision expressed by graduates varied considerably by EPA. The proportion of graduates rating themselves at expected level was high for “history taking”, “physical examination” “and documentation”; medium for “prioritizing differential diagnoses”, “interpreting results” and “developing and communicating a management plan”; low for “practical skills”; and very low for EPAs related to “urgent and emergency care”.

**Conclusions:**

Currently, there are significant gaps between the expectations of curriculum developers and the perceived competences of students. This is most obvious for practical skills and emergency situations. The new curriculum will either need to fill this gap or expectations might need to be revised.

**Supplementary Information:**

The online version contains supplementary material available at 10.1186/s12909-021-02534-w.

## Background

Experts regularly claim medical education to be outdated to prepare students for their profession as physicians [1]. This criticism has triggered a change towards competency-based medical education (CBME) incorporating outcome-based frameworks [2]. To implement competency-based curricula into clinical teaching, new teaching and assessment tools are needed [3]. Ten Cate’s idea of entrustment, first published in 2005 [4], has become popular and Entrustable Professional Activities (EPAs) have been adopted by many medical specialties. Initially predominantly used in postgraduate programs, there are sound arguments for their application in undergraduate medical education [5].

In 2017, Switzerland introduced a completely revised version of its national catalog for learning objectives for undergraduate medical training. The document is entitled “Principal Relevant Objectives and Framework for Integrated Learning and Education in Switzerland” (PROFILES) [6]. PROFILES is a prerequisite for the accreditation of undergraduate medical curricula in Switzerland and will define the content of the federal licensing exam as of 2021. The new catalog focuses on competency-based objectives. It is based on three pillars: general objectives related to the different role models of medical doctors (inspired by the Canadian Medical Education Directives for Specialists [CanMEDS] roles [7]), situations as starting points and EPAs. The Swiss initiative to implement end-of-training EPAs is derived from the Core-EPAs for Entering Residency published by the Association of American Colleges (AAMC) [8].

All Swiss universities providing undergraduate medical training are currently adapting their curricula to meet the new accreditation requirements. Among other things, PROFILES specifies that EPAs are “reflecting the main medical tasks that a physician must be able to perform autonomously on the first day of his residency” [6]. While such capabilities have always been the goal of undergraduate medical education, they are defined explicitly for the first time in Switzerland. The expected competency level is defined as Level 3: distant supervision.

We carried out an online survey with the 2019 graduates of the University of Zurich. These young physicians were trained following a pre-revision curriculum and passed the Federal Licensing Exam in accordance with the requirements of the predecessor of PROFILES, which is still in place. In this study, the participants evaluated their own levels of autonomy regarding EPAs as defined by PROFILES. In doing so, this study assesses how confident graduates feel to handle standard clinical situations and thus how well they feel prepared for clinical practice as defined by the new standard.

As the curricular reforms are currently being shaped and implemented, we aim at providing guidance to curriculum developers by clarifying which EPAs have already been covered adequately by current curricula and which need to be the focus of change. Furthermore, our data may serve as a baseline for examining future changes.

## Methods

In this section we describe our study design, the sampling and the data collection method.

### Assessing EPAs in a survey study

PROFILES lists nine main EPAs (see Table 1) derived from the Association of American Medical Colleges (AAMC) Core EPAs in the United States [8]. These are further specified into tasks (nested EPAs) and descriptors. This results in 161 items.

**Table 1:** Main EPAs as defined by PROFILES

EPA	Label
EPA 1	Take a medical history
EPA 2	Assess the physical and mental status of the patient
EPA 3	Prioritize a differential diagnosis following a clinical encounter
EPA 4	Recommend and interpret diagnostic and screening tests in common situations
EPA 5	Perform general procedures
EPA 6	Recognize a patient requiring urgent/emergency care; initiate evaluation and management
EPA 7	Develop a management plan; discuss orders and prescriptions in common situations
EPA 8	Document and present patient’s clinical encounter; perform handover
EPA 9	Contribute to a culture of safety and improvement

Designing a survey to evaluate students’ self-perception on the EPAs defined by PROFILES comes with several challenges. While the sheer length of the catalog is among them, the main concern is its heterogeneity. In accordance with a recent study by Meyer et al. [9], we found that some of the EPAs do not meet the standards of the EPA framework measured by the EQual rubric [10]. In this study, we aimed to only including high-quality EPAs. The list of EPAs was therefore edited for the purpose of this study. All authors are EPA experts. Three of the authors (AM, SF, SZ) hold a master’s degree in medical education and have been working with EPAs for several years. HBE is an educational expert and has gained extensive knowledge on the theory of EPAs while working on the implementation of PROFILES.

**Fig. 1:**
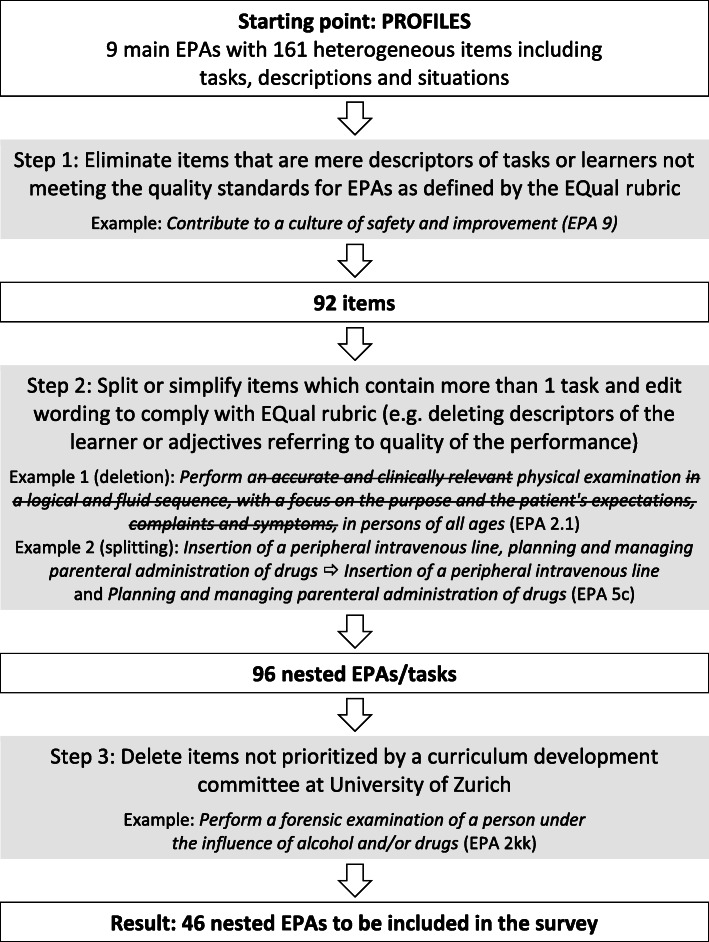
Selection and editing process for EPAs to be included in the survey

Figure 1 illustrates the selection and editing process. Each selection step was done by each of the authors separately; the resulting lists were then compared and discussed until consensus was reached. As a first step, items which were just descriptors of tasks and items that did not meet current EPA criteria [10] were discarded (e.g. all of EPA 9). As a second step, items that included more than one task were either split or simplified. Furthermore, the wording of the remaining tasks was edited to comply with the EPA framework; this resulted in 96 nested EPAs. As a final step, we compared our list with a prioritization previously done by a curriculum development committee of the Universities of Zurich. In a modified Delphi-process (not part of this study) the goal of this committee (consisting of two medical students, and 13 medical experts and faculty members) was to identify the Core-Tasks/EPAs within PROFILES. By deleting the EPAs not prioritized, a list of 46 items was generated.

When working with EPAs in medical education, competence is typically assessed using an entrustment-supervision scale [11]. However, PROFILES only includes the expected level: graduates are expected to handle all EPAs in the catalog “at least under distant, on-demand supervision on the first day of their residency” (p. 16) [12]. This corresponds to Level 3: “indirect, reactive supervision”.

Table 2 presents the wording used in our survey. We phrased the scale using the first person singular to make it more actionable for the participants. As we are focusing on undergraduate training, only Levels 1–3 are provided in our survey. The resulting questionnaire is included in the supplements.

**Table 2:** Entrustment levels used in the survey (derived from Ten Cate [11, 13])

Level	Label
1	I can only observe someone performing this task
2	I can perform the task under direct, proactive supervision (the supervisor is actively helping me)
3	I can perform the task under indirect, reactive supervision (I can ask for help and the supervisor is readily available, within a minute)^a^
4	I can perform the task under distant supervision (supervisor not present in the room; I can call the supervisor; supervisor available within 20–30 min)^b^
5	I can supervise others^b^

^a^ Level expected at end of medical school. ^b^ Level to be achieved during specialty training

### Sampling and data collection

An online survey was compiled using SurveyMonkey®. To minimize missing values, we used forced choice format for all questions considering EPAs. It was revised based on a pilot test with medical students and physicians working at the authors’ departments.

All 281 graduates of the Master of Human Medicine program of the University of Zurich who had passed the Federal Licensing Exam in September 2019 were invited to participate in the study by means of an email in October 2019. A reminder was sent after 13 and 24 days. Study participation was completely anonymous since IP addresses were not recorded and the software did not allow tracking of completed questionnaires to the originating email addresses. Participation in the study was voluntary and no financial or other incentive was offered. Once data collection was completed, the data were extracted from SurveyMonkey® and recorded in Microsoft Excel™ on a password-secured computer.

Ethical approval: the ethical commission of the Canton of Zurich has considered the project as not being within the scope of the Human Research Act (BASEC-Nr. Req-2019-00754).

## Results

Of 281 medical students who graduated in 2019, 152 completed the survey, resulting in a response rate of 54%. All analyses are based on these cases only. Average completion time was 8 min and 44 s. Mean age of participants was 26.7 years and 63% of the participants were female.

The need expressed by graduates for supervision varied considerably by EPA (the data are displayed in Table 3). For history taking (EPA 1) and documentation (EPA 8), the proportion of graduates rating themselves at Level 3 (indirect supervision) was high. For history taking, 99% of the graduates reported Level 3; for documentation in a patient’s chart and for providing oral presentations of patient encounters, the corresponding numbers were 84 and 86% respectively. For physical examination (EPA 2), the number of graduates rating themselves at Level 3 was high for general physical examination procedures but considerably lower for specific procedures, e.g. ophthalmological, dermatological and psychiatric examinations. Prioritizing differential diagnoses and interpreting results (EPAs 3 and 4) were regarded to be feasible at Level 3 for 61 and 65% of our respondents respectively. Developing and communicating a management plan (EPA 7) was deemed possible with indirect supervision by half of the respondents.

**Table 3:** Levels of autonomy reported by graduates for various tasks (percentages and absolute numbers)

EPA	Task (nested EPA, key referring to numbering in PROFILES)		Visualization of results^**a**^	Level of autonomy (in %, n in brackets)		
	Key	Description		Indirect, reactive supervision	Direct, proactive supervision	Observe only
1	1	Take a patient’s medical history (persons of all ages)	+++++	99% (150)	1% (2)	0% (0)
2	2.1	Perform a physical examination in persons of all ages	+++++	95% (145)	5% (7)	0% (0)
	2.2	Assess the cognitive and mental state of the patient including memory, perception, understanding, expression and affect	+++++	80% (122)	20% (30)	0% (0)
	2.5	Use devices such as stethoscope, otoscope and ophthalmoscope	+++++	90% (137)	9% (14)	1% (1)
	2.6	Explain physical examination maneuvers and obtain consent	+++++	90% (136)	9% (14)	1% (2)
	2a	Assessment of a patient’s general condition and vital signs	+++++	91% (138)	8% (12)	1% (2)
	2b	Assessment of a patient‘s nutritional status	++++	66% (100)	31% (47)	3% (5)
	2c	Assessment of a patient’s attention, thought, perception, speech, affect and psychomotor skills	++++	61% (92)	38% (58)	1% (2)
	2e	Assessment of skin, hair and nails, description of lesions	++++	61% (92)	38% (58)	1% (2)
	2f	Palpation of lymph nodes	+++++	93% (141)	6% (10)	1% (1)
	2j	Assessment of eye movements, recognition and description of nystagmus	++++	65% (99)	32% (49)	3% (4)
	2n	Inspection and palpation of thyroid, carotid arteries	+++++	81% (123)	18% (28)	1% (1)
	2o	Inspection and palpation of skeleton and joints	+++++	88% (134)	11% (16)	1% (2)
	2p	Functional testing of joint mobility: shoulders, elbows, wrists, fingers, hips, knees and ankles	+++++	83% (126)	16% (25)	1% (1)
	2q	Inspection, palpation, percussion and mobility of the spine	+++++	85% (129)	14% (21)	1% (2)
	2r	Inspection and palpation of chest, percussion and auscultation of lungs	+++++	98% (149)	1% (2)	1% (1)
	2 s	Palpation (apex beat/fremitus) and auscultation of heart; description of normal/abnormal heartbeat and murmurs	+++++	86% (130)	14% (22)	0% (0)
	2 t	Palpation of pulse, testing for arterial insufficiency or bruits	+++++	87% (133)	12% (18)	1% (1)
	2v	Assessment of venous system	++++	60% (91)	36% (55)	4% (6)
	2w	Palpation, percussion and auscultation of abdomen, description of findings	+++++	97% (148)	2% (3)	1% (1)
	2x	Inspection and palpation of groin/hernial orifices	+++	42% (64)	55% (83)	3% (5)
	2dd	Perform a neurological examination	+++++	90% (137)	10% (15)	0% (0)
	2ee	Assessment of coma (scale)	+++	56% (86)	39% (59)	5% (7)
3	3	Prioritize a differential diagnosis following a clinical encounter	++++	61% (93)	38% (57)	1% (2)
4	4	Recommend and interpret diagnostic and screening tests in common situations	++++	65% (99)	33% (50)	2% (3)
5	5a	Measuring and interpreting body temperature	+++++	97% (148)	2% (3)	1% (1)
	5b	Intravenous, subcutaneous and intramuscular injection	+++	56% (85)	41% (62)	3% (5)
	5c	Insertion of a peripheral intravenous line	+++	58% (88)	35% (54)	7% (10)
	5c	Planning and managing parenteral administration of drugs	++	21% (32)	55% (83)	24% (37)
	5f	Wound cleaning, application and removal of sutures	+++	43% (65)	47% (72)	10% (15)
	5 g	Application of bandages and dressings	++	36% (54)	54% (82)	10% (16)
	5o	Performance and interpretation of a urine stick test	++++	66% (101)	30% (45)	4% (6)
	5q	Performance and interpretation of an ECG	+++	51% (78)	46% (70)	3% (4)
	5r	Performance and interpretation of a pregnancy test	++++	74% (113)	21% (32)	5% (7)
6^b^	6a	Manage a patient with transient loss of consciousness, syncope, coma or seizures	+	18% (27)	66% (101)	16% (24)
	6b	Manage a patient with severe hypotension or shock	+	7% (11)	57% (86)	36% (55)
	6c	Manage a patient with acute chest pain	++	34% (52)	63% (95)	3% (5)
	6d	Manage a patient with acute severe headache or meningism	++	25% (38)	65% (99)	10% (15)
	6e	Manage a patient with acute abdominal pain	+++	45% (69)	52% (78)	3% (5)
	6 h	Manage a patient with severe hypertension	+	17% (26)	71% (108)	12% (18)
	6i	Manage a patient with uncomplicated trauma, such as a fall or minor traffic injury	++++	62% (94)	33% (51)	5% (7)
	6 k	Manage a patient with severe acute blood loss	+	9% (13)	54% (82)	37% (57)
7	7	Develop a management plan; discuss orders and prescriptions in common situations	+++	50% (76)	45% (69)	5% (7)
8	8.1	Document and record the patient’s chart	+++++	86% (130)	13% (20)	1% (2)
	8.3	Provide and incorporate a discharge document	++++	66% (100)	31% (48)	3% (4)
	8.5	Provide an oral presentation of a patient encounter and situation	+++++	84% (127)	14% (22)	2% (3)

All results are based on 152 cases

^a^ Visualization of proportion of respondents indicating requiring “indirect, reactive supervision” only: +++++ = 80–100%, ++++ = 60–79%, +++ = 40–59%, ++ = 20–39%, + = less than 20%

^b^ For EPA 6, the same three response levels were used as for the other EPAs even though PROFILES requires graduates to “autonomously and trustworthily manage [these situations] within the first 30 min” (p. 22) which refers to “distant supervision”

The responses for the practical skills listed in EPA 5 varied considerably by skill. Taking a patient’s temperature was regarded to be possible with indirect supervision by 97% of the graduates, whereas the corresponding figure was 21% for managing parenteral nutrition. The lowest levels of autonomy were reported for EPAs related to urgent and emergency care. While the variance for EPA 6 was very high, only one of eight items was regarded to be feasible with indirect supervision by the majority of the graduates. All other items ranged between 7 and 45%.

## Discussion

Our data show that graduates’ confidence in their abilities to perform the EPAs listed in the Swiss catalog of learning objectives (PROFILES) varies considerably by EPA. For some EPAs, most graduates regarded themselves as capable of performing the tasks at the expected level of autonomy, i.e. with indirect, reactive supervision. For other EPAs, only a few graduates felt confident to act with indirect supervision only.

Most participants felt confident to perform history taking, physical examination and documentation (EPAs 1, 2 and 8) with distant supervision. These are the mainstay of the medical profession and are taught early on. The importance of these skills is evident and the need to teach them at medical school has been emphasized in the literature [14–16]. Moreover, a lot of effort has recently been put into the qualitative assessment modalities of these medical skills – the emergence of OSCEs (Objective Structured Clinical Examination) being a good example [17]. These EPAs can also be taught, performed and assessed repeatedly in many clinical situations at a low risk for patients.

As expected, graduates feel less confident regarding specific physical examination techniques of smaller specialties. One example is EPA 2j: “Assessment of eye movements, recognition and description of nystagmus”. This might be due to the fewer contact hours these specialties are allocated throughout the curriculum. Ophthalmology, for example, occupies only 16 of the total 580 h of practical training in the Zurich curriculum. Only a few students acquire more ophthalmological experience during electives. Moreover, some of these skills are obviously difficult to study on models or in a simulation center.

The majority of graduates rated documentation (EPA 8) as feasible with indirect supervision. Again, these tasks are regularly performed by students during clerkships. However, specific teaching activities for documentation and handover skills do not (yet) exist in the Zurich medical curriculum. Therefore, these results are surprising as documentation, reporting and handovers are more complex than commonly perceived [18] and require formal teaching [19].

Students felt less confident regarding their capabilities for clinical reasoning without direct supervision, i.e. prioritizing differential diagnoses (EPA 3: 61%), recommending and interpreting tests (EPA 4: 65%) and developing management plans (EPA 7: 50%). On a positive note, very few participants suggested they should only observe (1–5%, depending on the EPA in question). This variance might be due to variation in the clinical situation respondents had in mind while working on the questionnaire. More importantly, these EPAs are very complex as they rely on the ability to integrate clinical data and subsequently to make sound choices. As studies have shown, clinical reasoning is not yet well-developed at the end of medical school; the skill of clinical reasoning grows with experience [20]. Therefore, the restrained confidence of our graduates is reasonable: experience is gained through repeated exposure to clinical situations and thus with time. Nendaz et al. described the crucial elements that foster clinical reasoning: active prior knowledge and use transfer between different cases; using both analytical and non-analytical skills; and emphasizing the systematic collection of clinical information [21]. Even though these tasks are part of a lifelong learning process, curricular reform should aim at integrating new teaching modalities facilitating these elements: flipped classroom models, problem-based learning or specific case-based teaching techniques to name a few [22]. More complex instructional design models such as the 4C/ID method (https://www.4cid.org/about-4cid) show the need for ways to integrate clinical reasoning in medical schools, but simple and short teaching elements such as “the one-minute preceptor” [23] are as good a tool in the teachers’ hands.

The results regarding procedural skills (EPA 5) are heterogeneous. This is not altogether surprising since the complexity of the assessed skills varies considerably. Tasks for which the respondents indicated particularly high levels of autonomy were taking a patient’s body temperature and performing a pregnancy test. Both tasks are simple and doable even for most of the general population without specific medical knowledge. Other EPAs pertain to classical medical procedural skills such as intravenous injection or insertion of a peripheral intravenous line. These skills are typical for the medical profession and taught during medical school. Many medical schools, Zurich among them, use simulation models in skills labs to teach these objectives. There is sound data that such training leads to immediate improvements when assessed in a simulated environment but transferability to clinical practice and retention of skills is less well understood [24]. The fact that less than 60% of our graduates regard themselves capable of performing the tasks mentioned under indirect supervision suggests that transferability is lacking and needs to be improved. No solution on how this is best achieved is to be found in the literature and further studies are much needed. As there is evidence that how much one practices matters [25, 26], curriculum developers and researchers need to consider the frequency of training opportunities.

Few surgical tasks are incorporated in the list of EPAs and, for these, only a few graduates rated themselves as competent to perform them with indirect supervision: 43 and 36% of graduates declared themselves comfortable with indirect supervision for wound cleaning and applying wound dressings respectively. A lack of surgical experience in undergraduate medical education has been previously reported [27]. Diminished exposure to certain specialties additionally poses a threat to the workforce as students may then not develop an interest in these specialties [28, 29].

EPAs covering emergency situations (EPA 6) reveal the largest gap between the self-assessed competences of graduates and the expectations put forth by PROFILES. Teaching and learning the correct management of medical emergency situations are considered to be among the most challenging issues even in postgraduate education [30–32]. The use of cognitive aids has been described as a means to increase performance in these stressful situations [33]. In only one of the eight acute care EPAs summarized in Category 6, more than 50% of graduates rated themselves as ready for indirect supervision, i.e. managing a patient with “uncomplicated trauma such as a fall or minor traffic injury”. This EPA differs from the others in Category 6 by being well-defined without possible variation in complexity. “Managing a patient with severe shock or acute blood loss” is considerably more complex. Less than 10% of the graduates rated themselves competent to take care of such patients with indirect supervision, while more than one in three graduates preferred to act with direct supervision. This might not necessarily imply lack of competence; rather, it might denote an awareness of the risk and complexity inherent in these possibly life-threatening situations. Students usually have limited exposure to emergency situations during undergraduate training. If they do, they rarely find themselves in the role of the team leader as, in emergencies; patient safety is usually prioritized over teaching. Therefore, we believe the self-assessments of our graduates to be adequate. This is further supported since there are data that self-assessment for these skills agrees well between trainees and supervisors [34]. The PROFILES expectation that graduates will be able to handle these emergency situations alone for 30 min is rather high. Our data indicate that graduates do not reach the expected degree of autonomy by far. If emergency situations are a priority, universities might want to increase learning opportunities, applying tools such as virtual reality, high-fidelity simulation and use of cognitive aids. Alternatively, national authorities need to lower the expected level of supervision for this section.

In summary, our study suggests that curriculum developers need to focus on clinical reasoning and clinical decision-making skills; they need to make sure rare but important procedural skills are not underrepresented and that students are frequently exposed to emergency situations even if they are simulated. Graduates feel adequately prepared for history taking, general clinical examination and documentation. Nevertheless, teaching these tasks should not be neglected.

There are some limitations to our study. First, the response rate raises the possibility of non-responder bias. Graduates willing to participate in this survey might belong to a specific subgroup and therefore self-selection might confound the data. As Saleh et al. summarized, response rates of email surveys have always been a limitation of method of data acquisition [35]. The rates were regarded as satisfying in the early 1990s with averages of 50%. Since then, they have declined greatly to numbers as low as 19% [36] which is believed to be due to the loss of novelty and survey-fatigue. In comparison, our response rate of 54% is high. Nevertheless, any curriculum developer following our recommendations needs to be aware of this bias.

Second, our data are subjective as they represent the self-assessments of graduates. Adding assessments by supervisors might have increased the validity of the dataset since some authors have shown that students overestimate their competence in practical clinical skills [37]. On the other hand, there is evidence that self-assessment using entrustment-supervision scales seems to be accurate [34].

Third, the wording of the EPA leaves room for interpretation. The more specific the title of an EPA, the easier it is for the trainees to rate themselves. Many EPA titles in PROFILES are not specific and trainees’ perception of them (e.g. their complexity) might differ. This might lead to different self-assessment.

Fourth, generalizability might be limited because we have only evaluated data from one university and one cohort. However, the results are not surprising and are in agreement with previous research. We are therefore confident that the data are meaningful for curriculum developers at other institutions as well.

Lastly, we only focused on self-evaluations of undergraduate medical students. Since PROFILES and EPAs are yet to be implemented, no evaluations by clinical supervisors were available. To us it was important to give students a voice. We are planning a follow-up study which will include a comparison between supervisors and medical students’ evaluation of EPAs. Studies, including the faculty’s assessment of new residents, are necessary to evaluate the results effected by the curriculum change objectively.

## Conclusion

As defined in PROFILES, all EPAs should be mastered at Level 3, i.e. with indirect supervision. However, our study reveals a substantial gap between this expectation and the self-reported level of competence of current graduates. Graduates indicated that they were well-prepared for low-risk tasks. On the other hand, they felt less prepared for high-risk tasks, such as performing procedural skills or handling emergency situations. This study provides important information for curriculum reforms: it reveals areas where reform is much needed and areas already well-covered by the current curriculum in medical school. Depending on the political commitment to provide resources for medical education, medical schools need to decide on how to allocate funding and teaching time.

## Supplementary Information

**Additional file 1.** Supplement 1: Questionnaire.

## Data Availability

The datasets used and/or analysed during the current study are available from the corresponding author on reasonable request.

## Abbreviations

AAMC: Association of American Medical Colleges
CanMEDS: Canadian Medical Education Directives for Specialists
CBME: Competency-Based Medical Education
EPA: Entrustable Professional Activity
OSCE: Objective Structured Clinical Examination
PROFILES: Principal Relevant Objectives and Framework for Integrated Learning and Education in Switzerland
