# Supplementary material for: An analysis of core EPAs reveals a gap between curricular expectations and medical school graduates’ self-perceived level of competence
Source: BMC Med Educ. 2021 Feb 16;21:105. doi: 10.1186/s12909-021-02534-w (PMC7885554; doi:10.1186/s12909-021-02534-w)
Supplement: Supplementary file 1 — Additional file 1. Supplement 1: Questionnaire. [file 12909_2021_2534_MOESM1_ESM.docx]

Supplement 1: Questionnaire

**Title: Competence at the end of Medical School**

Question 1:

**Important note: For all the following questions, we use the same 3-point rating scale:

1. Observe:** you can just observe somebody perform the task. This is the default mode if the other two don't apply.
**2. Direct supervision:** you are able to perform the task with the supervisor present and proactively helping you.
**3. Distant, on-demand supervision:** you are able to perform the task independently, but you can call the supervisor and she/he is readily available (=reactive/indirect supervision)
 **Please confirm that you understand this rating scale.**

| OK, got it! |
| --- |

Question 2:

**Section 1: Take a medical history Please rate to the best of your abilities. How much supervision would you need to perform the following task?**

| 1. Take a patient’s medical history (persons of all ages) |
| --- |

Question 3:

**Section 2: Assess the physical and mental status of the patient Please rate to the best of your abilities. How much supervision would you need to perform the following tasks?**

| 1. Perform physical examination in persons of all ages |
| --- |
| 2. Assess the cognitive and mental state of the patient including memory, perception, understanding, expression and affect |
| 3. Use devices such as stethoscope, otoscope, ophthalmoscope |
| 4. Explain physical examination manoeuvres and obtain consent |
| 5. Assessment of a patient’s general condition and vital signs |
| 6. Assessment of a patient‘s nutritional status |
| 7. Assessment of a patient’s attention, thought, perception, speech, affect and psychomotor skills |
| 8. Assessment of skin, hair and nails, description of lesions |
| 9. Palpation of lymph nodes |
| 10. Assessment of eye movements, recognition and description of nystagmus |
| 11. Inspection and palpation of thyroid, carotid arteries |
| 12. Inspection and palpation of skeleton and joints |
| 13. Functional testing of joint mobility: shoulders, elbows, wrists, fingers, hips, knees and ankles |
| 14. Inspection, palpation, percussion and mobility of the spine |
| 15. Inspection and palpation of chest, percussion and auscultation of lungs |
| 16. Palpation (apex beat/fremitus) and auscultation of heart; description of normal/abnormal heartbeat and murmurs |
| 17. Palpation of pulse, testing for arterial insufficiency or bruits |
| 18. Assessment of venous system |
| 19. Palpation, percussion and auscultation of abdomen, description of findings |
| 20. Inspection and palpation of groin / hernial orifices |
| 21. Perform a neurological examination |
| 22. Assessment of coma (scale) |

Question 4:

**Section 3: Prioritize a differential diagnosis following a clinical encounter Please rate to the best of your abilities. How much supervision would you need to perform the following task?**

| 1. Prioritize a differential diagnosis following a clinical encounter |
| --- |

Question 5:

**Section 4: Recommend and interpret diagnostic and screening tests in common situations. Please rate to the best of your abilities. How much supervision would you need to perform the following task?**

| 1. Recommend and interpret diagnostic and screening tests in common situations |
| --- |

Question 6:

**Section 5: Perform general procedures. Please rate to the best of your abilities. How much supervision would you need to perform the following tasks?**

| 1. Measuring and interpreting body temperature |
| --- |
| 2. Intravenous, subcutaneous and intramuscular injection |
| 3. Insertion of a peripheral intravenous line |
| 4. Planning and managing parenteral administration of drugs |
| 5. Wound cleaning, application and removal of sutures |
| 6. Application of bandages and dressings |
| 7. Performance and interpretation of a urine stick test |
| 8. Performance and interpretation of an ECG |
| 9. Performance and interpretation of a pregnancy test |

Question 7:

**Section 6: Recognize a patient requiring urgent / emergency care, initiate evaluation and management. Please rate to the best of your abilities. How much supervision would you need to perform the following tasks?--> i.e. assess the patient’s state, order and interpret tests, initiate procedures and treatment**

| 1. Manage a patient with transient loss of consciousness, syncope, coma, or seizures |
| --- |
| 2. Manage a patient with severe hypotension or shock |
| 3. Manage a patient with acute chest pain |
| 4. Manage a patient with acute severe headache or meningism |
| 5. Manage a patient with acute abdominal pain |
| 6. Manage a patient with severe hypertension |
| 7. Manage a patient with uncomplicated trauma such as fall, minor traffic injury |
| 8. Manage a patient with severe acute blood loss |

Question 8:

**Section 7: Develop a management plan, discuss orders and prescriptions in common situations. Please rate to the best of your abilities. How much supervision would you need to perform the following task?**

| 1. Develop a management plan, discuss orders and prescriptions in common situations |
| --- |

Question 9:

**Section 8: Document and present patient’s clinical encounter; perform handover. Please rate to the best of your abilities. How much supervision would you need to perform the following tasks?**

| 1. Document and record the patient’s chart |
| --- |
| 2. Provide and incorporate a discharge document |
| 3. Provide an oral presentation of a patient encounter and situation |

Question 10:

**What is your age? (in years...)**

Question 11:

**Gender?**

| Female |
| --- |
| Male |
| No Answer |

Question 12:

**What specialties have you worked in for more than 1 month (e.g. as )? (more than one answer possible)**

| Anaesthesiology |
| --- |
| General Practitioner |
| Internal Medicine |
| Paediatrics |
| Surgery |
| Other (we are eager to know what...) |

Question 13:

**If you had to choose today: what kind of specialist do you want to become?**

| Anaesthesiology |
| --- |
| General Practitioner |
| Internal Medicine |
| Paediatrics |
| Surgery |
| Other (we are eager to know what...) |

Question 14:

**Besides being a medical student, do you have other job experience?**

| No |
| --- |
| Yes! (please let us know what...) |

Question 15:

**If you should have any additional thoughts and comments, please let us know here.**

|  |
| --- |

**Thank you very much for your participation!
We appreciate it very much.
Good luck with your plans!**
